# Parenteral administration of factor Xa/IIa inhibitors limits experimental aortic aneurysm and atherosclerosis

**DOI:** 10.1038/srep43079

**Published:** 2017-02-21

**Authors:** Corey S. Moran, Sai-Wang Seto, Smriti M. Krishna, Surabhi Sharma, Roby J. Jose, Erik Biros, Yutang Wang, Susan K. Morton, Jonathan Golledge

**Affiliations:** 1 Queensland Research Centre for Peripheral Vascular Disease, College of Medicine and Dentistry, James Cook University, Townsville, 4811, Australia; 2 National Institute of Complementary Medicine, Western Sydney University, Campbelltown, 2560, Australia; 3 School of Applied and Biomedical Sciences, Faculty of Science and Technology, Federation University Australia, Mount Helen, 3350, Australia; 4 Department of Vascular and Endovascular Surgery, The Townsville Hospital, Townsville, 4814, Australia

## Abstract

Intraluminal thrombus is a consistent feature of human abdominal aortic aneurysm (AAA). Coagulation factor Xa (FXa) catalyses FII to thrombin (FIIa). We examined the effect of FXa/FIIa inhibition on experimental aortic aneurysm in apolipoprotein E-deficient (ApoE^−/−^) mice infused with angiotensin II (AngII). The concentration of FXa within the supra-renal aorta (SRA) correlated positively with SRA diameter. Parenteral administration of enoxaparin (FXa/IIa inhibitor) and fondaparinux (FXa inhibitor) over 14 days reduced to severity of aortic aneurysm and atherosclerosis in AngII-infused ApoE^−/−^ mice. Enteral administration of the FIIa inhibitor dabigatran had no significant effect. Aortic protease-activated receptor (PAR)-2 expression increased in response to AngII infusion. Fondaparinux reduced SRA levels of FXa, FIIa, PAR-2, matrix metalloproteinase (MMP)2, Smad2/3 phosphorylation, and MOMA-2 positive cells in the mouse model. FXa stimulated Smad2/3 phosphorylation and MMP2 expression in aortic vascular smooth muscle cells (VSMC) *in vitro.* Expression of MMP2 in FXa-stimulated VSMC was downregulated in the presence of a PAR-2 but not a PAR-1 inhibitor. These findings suggest that FXa/FIIa inhibition limits aortic aneurysm and atherosclerosis severity due to down-regulation of vascular PAR-2-mediated Smad2/3 signalling and MMP2 expression. Inhibition of FXa/FIIa may be a potential therapy for limiting aortic aneurysm.

Abdominal aortic aneurysm (AAA) is an age related degenerative disease which is present in ~5% of men and ~1% of women aged ≥ 65 years and is an important cause of sudden death in older adults due to aortic rupture or associated athero-thrombotic events^1^. With the introduction of screening programs and the common use of abdominal imaging most AAAs are detected at a small size. Approximately 50% of small AAA (<55 mm) eventually expand to a size where surgical intervention is required to prevent rupture^1^. There is currently no effective drug intervention to limit AAA growth^2^.

Activation of the coagulation cascade has long been implicated in cardiovascular events such as acute coronary syndrome^3^,^4^. Anti-coagulant therapies are an important component of management of such events but are not routinely indicated in patients that have AAAs. Most human AAAs contain significant amounts of intraluminal thrombus (ILT), the volume of which has been shown to correlate strongly with AAA size and growth^5^,^6^. Human AAA thrombus contains high concentrations of pro-inflammatory cytokines, chemokines, inflammatory cells and proteolytic enzymes which are all implicated in AAA pathogenesis^7^,^8^. Constant remodelling of the thrombus involving fibrin deposition and degradation is implicated in promoting aortic wall inflammation^9^.

Factor Xa (FXa) plays a central role in the coagulation cascade acting as the convergence point of the intrinsic and extrinsic coagulation pathways and catalyses the conversion of factor II (FII) to factor IIa (FIIa)^10^. Recent studies have suggested that both FXa and FIIa have a direct role in the pathogenesis of vascular diseases^11^,^12^. A number of studies have suggested that inhibition of these clotting factors may reduce atherosclerosis progression in mouse models^13^,^14^,^15^,^16^,^17^,^18^,^19^, however the effect of anticoagulants on AAA progression has not been explored.

Direct cellular effects of FXa on the vasculature are mediated by the G-protein coupled protease-activated receptors (PAR), PAR-1 and PAR-2^20^,^21^,^22^. Activation of these receptors promotes various cellular responses, such as smooth muscle cell migration, vascular remodelling, angiogenesis and inflammation, which are implicated in atherosclerosis and aneurysm pathogenesis. Expression of PAR-2 has been shown to be increased in rodent models of vascular injury and human atherosclerosis^23^,^24^,^25^. FXa has been shown to induce PAR-2, but not PAR-1 expression, via a FIIa independent mechanism in human vascular smooth muscle cells, suggesting an independent role of FXa on PAR-2 signalling^26^. Activation of the Smad signalling pathway by FXa has been suggested as a potential therapeutic target for anticoagulants^27^, although the contribution of PAR-mediated Smad signalling, stimulated by FXa, to AAA and atherosclerosis pathogenesis has not been studied.

We administered enoxaparin (a low molecular weight heparin), fondaparinux (a synthetic, selective FXa inhibitor) or dabigatran (a direct FIIa inhibitor) to angiotensin II (AngII)-infused apolipoprotein E deficient (ApoE^−/−^) mice to assess the effect of anticoagulation therapy in an experimental model of aortic aneurysm that incorporates thrombus formation and atherosclerosis.

## Results

### AAA severity within the ApoE^−/−^ mice infused with AngII correlated positively with aortic concentration of FXa

**Figure 1:**
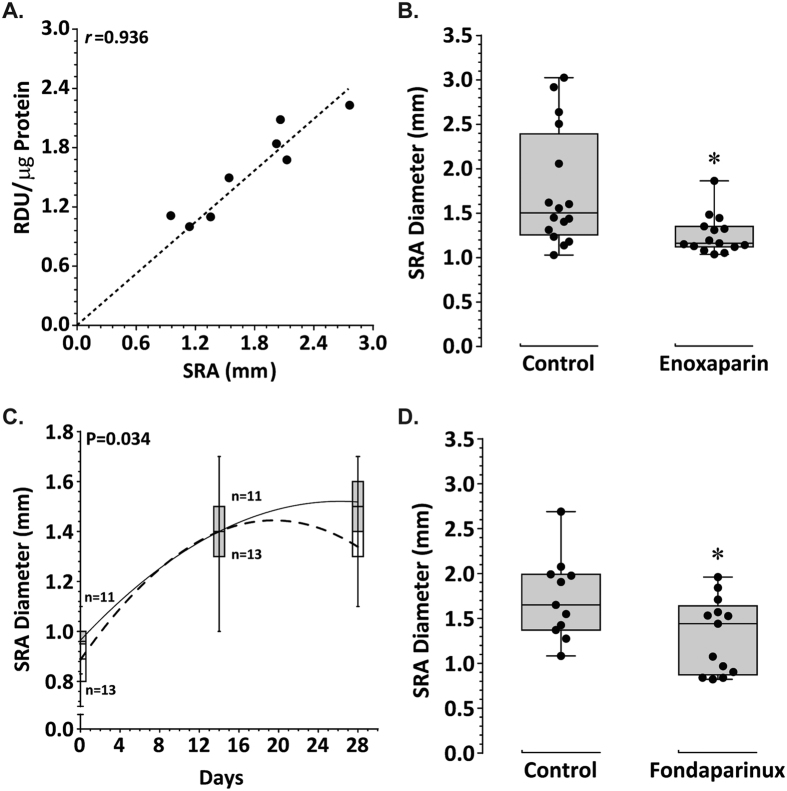
Effect of enoxaparin and fondaparinux on AngII-induced aortic dilatation in *ApoE*^−/−^mice. (**A**) Relationship demonstrated between increasing SRA diameter and SRA concentration of FXa by Pearson correlation (r) coefficient; P = 0.001. (**B**) Maximum SRA diameter determined by morphometry at study end (day 28) in ApoE^−/−^ mice receiving vehicle (control, n = 16) or enoxaparin (2.0 mg/kg/48 hrs, s.c., n = 16) for 14 days. Data expressed as median and interquartile range (mm) with maximum and minimum data points (whiskers); *P = 0.008 by Mann-Whitney U test. (**C**) SRA diameter measured by ultrasound in mice receiving vehicle (solid line/grey bar) or fondaparinux (300 μg/kg/day, s.c.; dashed line/white bar). Data expressed as median and interquartile range with maximum and minimum data points (whiskers); P = 0.034 for difference between groups by linear mixed-effects. (**D**) Maximum SRA diameter at study end (day 28) in ApoE^−/−^ mice receiving vehicle (control, n = 11) or fondaparinux (300 μg/kg/day, s.c, n = 13) for 14 days. Data expressed as median and interquartile range with maximum and minimum data points (whiskers); *P = 0.028 by Mann-Whitney U test.

AngII infusion induces aortic aneurysm formation in ApoE^−/−^ mice with the suprarenal aorta (SRA) being the common aortic site affected. FXa was abundantly expressed on Western blotting of proteins from large AAAs (Fig. S1). There was a strong positive correlation between the aortic levels of FXa and SRA diameter (*r* = 0.936, P = 0.001; Fig. 1A).

### Reduction of aortic aneurysm severity in mice administered enoxaparin

Enoxaparin (or vehicle) was administered via subcutaneous injection 14 days after commencement of AngII infusion and continued daily for the remainder of the infusion period. There were five fatalities due to aortic rupture in each of the two groups during the 14-day treatment period (see Supplementary Fig. S2). These aortas were successfully retrieved and included in final analyses. Administration of enoxaparin was associated with a significant decrease in aortic FXa activity compared to vehicle (0.004 ± 0.001 ng/μg protein versus 0.65 ± 0.14 ng/μg protein, respectively; P < 0.001; see Supplementary Fig. S3). Median maximum diameter of the SRA was significantly smaller in mice receiving enoxaparin compared to vehicle (Fig. 1B).

### Reduction of aortic aneurysm severity in mice administered fondaparinux

**Figure 2:**
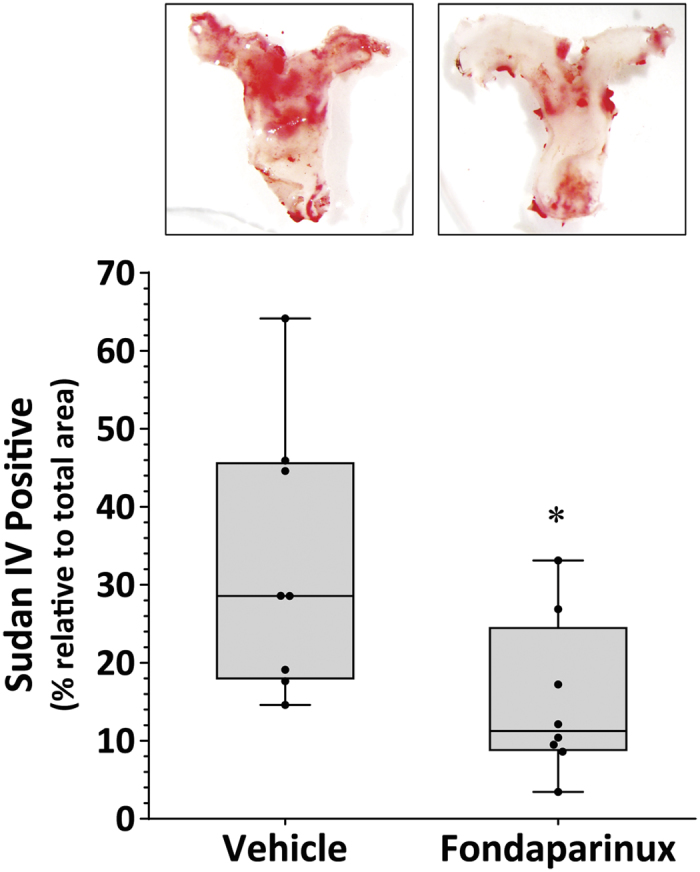
Reduced aortic arch atheroma in AngII-infused ApoE^−/−^ mice administered fondaparinux. Reduced Sudan IV staining within the aortic arch of mice receiving fondaparinux (300 μg/kg/day, s.c.) compared to vehicle. Data expressed as median and interquartile range with maximum and minimum data points (whiskers) for positive staining area relative to total specimen area (%); *P = 0.025 compared by Mann-Whitney U test; n = 8.

Fondaparinux (or vehicle) was administered via subcutaneous injection 14 days after commencement of AngII infusion and continued daily for the remainder of the infusion period. Ultrasound measurements demonstrated that SRA expansion in response to AngII was reduced in mice receiving fondaparinux compared to controls (P = 0.034) (Fig. 1C). Aortas from all mice were harvested at the end of the study. Administration of fondaparinux significantly reduced the median level of aortic FXa compared to vehicle (0.77, 0.61–0.88 RDU/μg protein vs 1.12, 1.01–1.21 RDU/μg protein; P = 0.008; Supplementary Fig. S3). The concentration of FIIa within the aorta was also reduced in mice receiving fondaparinux (1.37, 0.82–1.53 RDU/μg protein vs 1.87, 1.72–1.91 RDU/μg protein, respectively; P = 0.008; Supplementary Fig. S3). The median maximum SRA diameter in mice receiving fondaparinux was significantly reduced in comparison to controls (Fig. 1D). Intimal atherosclerotic lesion area within the aortic arch was assessed using Sudan IV staining. Mice receiving fondaparinux had significantly less mean positive Sudan IV staining area compared to the vehicle group (P = 0.025; Fig. 2).

### Limited effect of oral administration of dabigatran on aortic aneurysm severity

Dabigatran etexilate (DE) was administered via medicated chow initiated 14 days after commencement of AngII infusion. Median concentration of FIIa within the aorta was reduced in mice receiving DE compared to controls (1.12, 1.00–1.85 RDU/μg protein vs 2.18, 2.07–2.24 RDU/μg protein, respectively; P = 0.032; Supplementary Fig. S3). Median maximum SRA diameter remained comparable between mice receiving DE and mice receiving control chow throughout the intervention period as assessed by ultrasound (Supplementary Fig. S4), and confirmed by morphometric analysis at the end of the study (1.54, 1.32–1.95 mm vs 1.62, 1.37–2.38 mm, respectively; P = 0.684). Aortic arch Sudan IV staining area was smaller in mice administered dabigatran compared with mice receiving vehicle, however statistical significance was not demonstrated (16.95 ± 7.96% vs 20.30 ± 12.65%, respectively; n = 6; P = 0.474).

### MMP2 and PAR-2 are downregulated within the SRA of mice administered fondaparinux

AngII infusion resulted in higher levels of PAR-2 protein within the SRA of ApoE^−/−^ mice compared to saline-infused controls (median relative expression 1.262, inter-quartile range 1.108–1.339, n = 6 versus 0.742, 0.560–0.815, n = 6, respectively, relative to Gapdh; P = 0.002). Gene expression for PAR-2 and MMP2 within the SRA of mice receiving fondaparinux was down-regulated 3- and 12-fold, respectively, compared to vehicle (Table 1). mRNA for MMP2 was reduced in mice receiving DE compared to vehicle but the difference was not statistically significant (Table 1). Gene expression for PAR-1 and MMP9 within the SRA was similar in mice receiving vehicle, fondaparinux, and DE (Table 1).

### Fondaparinux reduced AngII-induced elastin degradation and inflammation within the aorta

**Figure 3:**
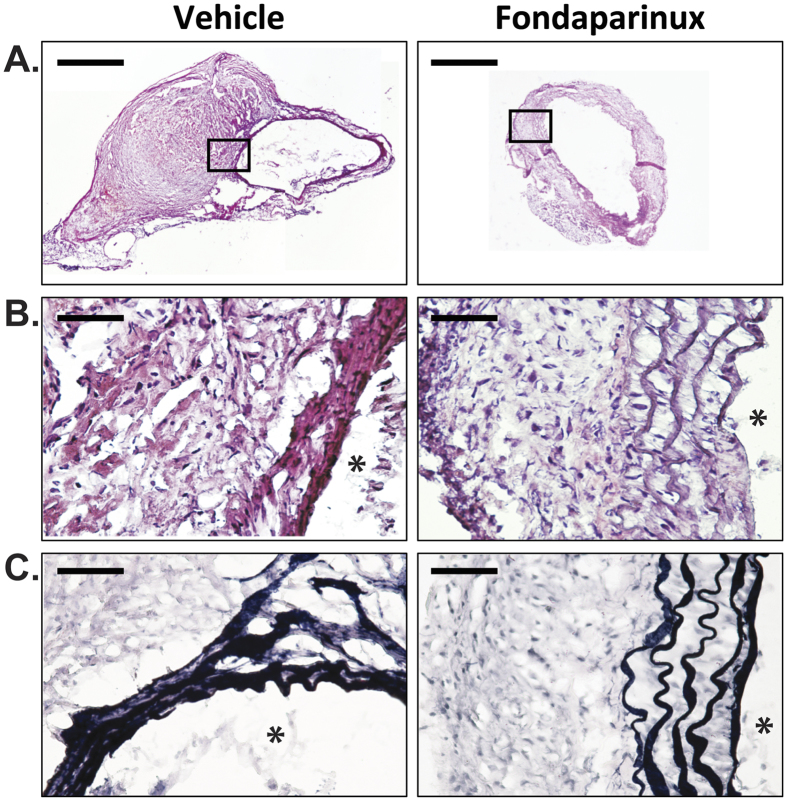
Effect of fondaparinux on remodelling within the aortic wall in response to AngII. (**A**) H&E micrographs at low magnification (scale bar 500 μm) of whole transverse section through the SRA showing smaller aortic diameter in mice administered fondaparinux (300 μg/kg/day, s.c.) compared to vehicle for 14 days associated with reduced presence of intramural thrombus. The region within the black box corresponds to the magnified fields shown in figures **B** and **C**. (**B**) Preserved lamellar pattern of aortic elastin (black after staining with the Verhoeff-Van Giesson, (**C**) in mice administered fondaparinux (300 μg/kg/day, s.c.) compared to vehicle for 14 days (scale bar 50 μm); *lumen.

**Figure 4:**
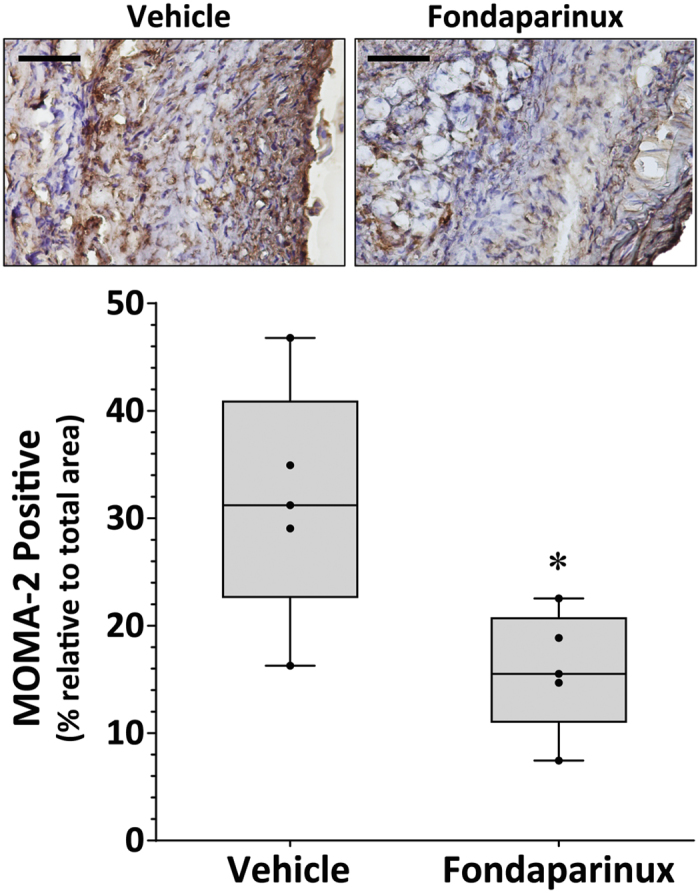
Fondaparinux down-regulated inflammation within the aortic wall in response to AngII. Staining for MOMA-2 (monocyte/macrophage) within the SRA of AngII-infused ApoE^−/−^ mice receiving fondaparinux (300 μg/kg/day, s.c.) compared to vehicle control for 14 days. Shown are data expressed as median and interquartile range with maximum and minimum data points (whiskers) for positive staining area relative to total specimen area (%); *P = 0.032 by Mann-Whitney U test; n = 5 (scale bar 50 μm).

Intramural thrombus formation within the SRA appeared to be reduced in mice administered fondaparinux compared to vehicle upon qualitative histological assessment (Fig. 3A). Elastin fibre fragmentation within the SRA also appeared less severe in mice receiving fondaparinux (Fig. 3B and C). The median ratio of aortic phosphorylated-to-total smad2/3 was significantly reduced in mice receiving fondaparinux compared to vehicle (2.43, 2.27–2.55 vs 3.11, 3.10–3.58, respectively; P = 0.008; see Supplementary Fig. S5). Immunostaining for monocyte/macrophages (MOMA-2) within the adventitia and media of the SRA was significantly reduced in mice administered fondaparinux compared to vehicle (P = 0.032; Fig. 4).

### Smad phosphorylation and upregulation of MMP2 in VSMC by FXa via PAR-2 *in vitro*

**Figure 5:**
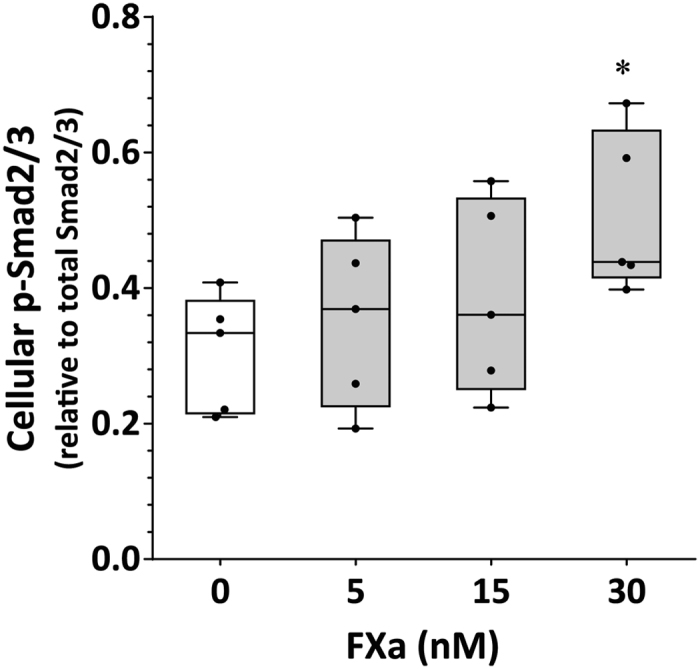
Effect of FXa on Smad2/3 phosphorylation in human aortic VSMCs *in vitro*. Dose-dependent increase in phosphorylated/total ratio for Smad2/3 in VSMCs exposed to increasing concentrations of FXa over 24 hours. Data expressed as median and interquartile range with maximum and minimum data points (whiskers) for p-Smad2/3 relative to total Smad2/3 protein; *P < 0.05 by Kruskal-Wallis test with Dunn’s multiple comparisons post-hoc; n = 5.

Incubation of human aortic VSMC with FXa (5–30 nM) induced a concentration-dependent increase in cellular Smad2/3 phosphorylation over 24 hours (Fig. 5; Supplementary Fig. S5). Smad2/3 phosphorylation induced by FXa (30 nM) and Smad2/3 mRNA were down-regulated upon inhibition of PAR-2 (FSLLRY-NH_2_; 10 μM), but were not affected in the presence of a PAR-1 antagonist (SCH79797; 10 μM) (Table 2; Supplementary Fig. S5). Exposure of VSMC to FXa induced an increase in median mRNA expression of MMP2 (relative to GAPDH) from 0.57, 0.49–0.64 to 0.86, 0.75–1.03 (P = 0.004). No significant change in mRNA expression for MMP9 was observed (0.58, 0.50–0.64 vs 0.63, 0.57–0.75; P = 0.238). Co-incubation of FXa-stimulated VSMC with the PAR-2 antagonist inhibited the up-regulation of MMP2 mRNA resulting in reduced supernatant levels of the protein (Table 2; see Supplementary Fig. S6). No effect on MMP2 mRNA or protein was observed in FXa-stimulated cells incubated in the presence of the PAR-1 antagonist (Table 2; Supplementary Fig. S6).

## Discussion

Intra-luminal thrombus (ILT) is present in most human AAAs and more rapid AAA growth is associated with larger ILT volume^5^,^6^. Although, the role of ILT in AAA is controversial, recent studies suggest that AAA thrombus is an important source of pro-inflammatory cytokines, reactive oxygen species and proteases implicated in AAA growth^7^,^8^,^9^,^28^. Circulating concentrations of thrombin-antithrombin III complex, D-dimer, and several clotting factors are elevated in AAA patients^2^,^29^. We examined the effect of the anticoagulants enoxaparin, fondaparinux, and dabigatran on growth of experimental aortic aneurysm in the AngII-infused ApoE^−/−^ mouse model. Our results suggest that inhibition of FXa/FIIa limits the severity of aortic aneurysm and atherosclerosis in this model.

The presence of thrombus within the aortic wall is often seen in aortic aneurysms induced by AngII in the ApoE^−/−^ mouse^30^. Development of intra-mural haematoma in this mouse model follows aortic dissection and a bleed into the vessel wall^31^, thus the presence of clotting factors within the aortic wall may not be unexpected. Enoxaparin is a low molecular weight heparin that complexes with anti-thrombin III (ATIII) to neutralise FXa, and, to a lesser degree, FIIa activity^32^. Administration of enoxaparin in the current mouse model significantly reduced FXa activity within the aorta, although it is likely that inhibition of both FXa and FIIa activity contributed to the markedly decreased aortic response to AngII, suggesting that clotting factor activity within the aortic wall supported aortic dilatation in this model rather than being a consequence of it.

Factor IIa is produced by the enzymatic cleavage of prothrombin by FXa^10^. Unlike enoxaparin, the smaller low molecular weight heparin, fondaparinux, via complex with ATIII inactivates FXa but not FIIa^33^, thereby selectively inhibiting FIIa formation rather than FIIa activity. The efficacy of fondaparinux in our model was demonstrated with reduced levels of FIIa measured within the SRA of mice administered fondaparinux compared to control. The inhibition of AngII-induced aortic aneurysm in mice receiving fondaparinux therefore indicates an importance for local FIIa production within the aortic wall (and subsequent activity) in promoting aortic aneurysm development in this model.

A subsequent study was performed with the selective FIIa inhibitor, dabigatran. Unexpectedly, the administration of dabigatran did not have a significant effect on aneurysm growth. Dabigatran is orally available as the pro-drug dabigatran etexilate (DE)^34^, administered in the current study via medicated chow. The dose and administration regimen used has been reported to limit atherosclerosis successfully in other models and we confirmed that DE administration reduced aortic concentration of FIIa. However we were not able to assess the effect of DE on other markers of coagulation. It is therefore possible that dabigatran levels may have been too low to be effective. The DE-supplemented chow used in the current study contained the same dose previously reported to be effective at producing anticoagulant effects in other rodent models^13^,^14^,^15^,^16^,^17^ and was recommended and supplied by the manufacturer (Boehringer Ingelheim Pharma GmbH & Co KG, Germany). Moreover, some effects of DE administration were demonstrated. For instance, FIIa protein was significantly lower in aortic tissue from mice administered DE compared to mice on control diet. These outcomes provide evidence for the action of DE in the current mouse model.

It is important to understand that the model used in the current study differed significantly from previous investigations of DE, in that it incorporated established vascular disease in which both aortic aneurysm and atherosclerosis were induced prior to the commencement of drug administration. The subcutaneous infusion of AngII accelerates progression and severity of atherosclerosis and aneurysm within the aorta of ApoE^−/−^ mice fed either a standard or high-fat diet^30^. Previous studies investigating DE in the ApoE^−/−^ model^13^,^14^,^15^,^16^,^17^ have done so without AngII infusion. The administration period for DE in the current study was 14 days, with the intervention initiated 14 days after commencement of AngII infusion. In previous studies, DE has been administered at the start of the experiment and continued throughout an extended experimental period of 28–84 days^13^,^14^,^15^,^16^,^17^. Finally, the mode of DE administration (enteral) was different to that for enoxaparin and fondaparinux (parenteral). These differences in study design from previous investigations likely explain our observations.

Based on our mouse model findings, further investigations were focused upon potential mechanisms by which fondaparinux inhibited aortic dilatation. The aetiology of AAA is multifactorial with a chronic aortic inflammatory response considered central to AAA pathogenesis^35^. Recent evidence supports a significant role of innate immune cells, particularly monocyte/macrophages, in AAA pathogenesis^36^,^37^. In the current study, the presence of monocyte/macrophages within aortas of mice administered fondaparinux appeared reduced compared to aortas of control mice, suggesting that aortic infiltration by these cells was associated with FXa activity within the aortic wall. Indeed, FXa-stimulated Smad phosphorylation has been implicated in promoting inflammation, matrix remodelling and AAA^27^,^38^. In this context, the current finding that aortic Smad2/3 activity was reduced in AngII-infused mice administered fondaparinux is potentially important.

Studies have suggested that cellular responses to FXa involve the activation of PARs^39^. Most evidence suggests PAR-2 activation mediates the vascular effects of FXa^40^. Expression of PAR-2 has been shown to be markedly increased in rodent models of vascular remodelling and human atherosclerosis^23^,^24^,^25^. Moreover, FXa induces PAR-2 expression in human VSMCs and has been reported to enhance PAR-2-mediated cellular oxidative stress and inflammation in vessel injury models^26^. Here we report increased expression of PAR-2 within aortic tissue of ApoE^−/−^ mice in response to AngII and that FXa-induced Smad2/3 phosphorylation (activity) in VSMC *in vitro* is PAR-2 sensitive. In addition, PAR-2 gene expression within the SRA of AngII-infused mice was down-regulated with the administration of fondaparinux. Together, these data suggest a potential mechanism by which fondaparinux acted to suppress an AngII-induced aortic inflammatory response, via inhibition of FXa-mediated activation of PAR-2 and down-regulation of Smad2/3.

Upregulation of proteolytic activity within the aorta is believed to be critical in the degeneration of medial architecture associated with advanced-stage human AAA^41^. Compromised matrix repair in the damaged aorta associated with the loss of synthetic capability of VSMC due to apoptosis is believed to be important in the evolution of aneurysm degeneration^42^. There is evidence however that aortic VSMCs have the potential to directly participate in the degenerative process^43^,^44^. Human AAA tissue-derived VSMCs exhibit increased elastolytic activity compared to VSMCs from non-diseased aortas^44^. VSMC-derived MMP-2 has been particularly implicated in AAA^45^. Qualitative histological examination of aortic tissue from our mouse model suggested a more conserved structure of elastin fibres within the aorta of mice administered fondaparinux compared to vehicle, which may be associated with the marked down-regulation in MMP2 gene expression within the artery. Our *in vitro* studies demonstrated that exposure of healthy human VSMCs to FXa resulted in the upregulation of MMP-2, an effect abrogated in the presence of a PAR-2, but not a PAR-1, antagonist, thus linking FXa-mediated PAR-2 activation with matrix remodelling within the aneurysmal aorta.

Several limitations to this study are highlighted. First, suitably prepared plasma samples for coagulation tests were not available. The value of functional tests for coagulation to assess effective delivery of anticoagulants *in vivo* is acknowledged. While a significant effect on aneurysm growth was not observed with direct inhibition of FIIa by dabigatran, the authors accept that without plasma data for the anticoagulants and coagulation tests, conclusions around dabigatran efficacy and the importance of FIIa to aortic dilatation in the current model are uncertain. Nevertheless, it is reasonable to assume that reduced FIIa formation within the aortic wall consequent to FXa neutralisation is an important factor in the inhibition of aortic aneurysm growth by enoxaparin and fondaparinux clearly demonstrated in our mouse model. Second, only one *in vivo* model of AAA was investigated; however, we believe that the AngII-ApoE^−/−^ mouse model is more representative of the clinical situation of established vascular disease in that it is model of aortic aneurysm that incorporates thrombus formation and atherosclerosis. Third, the current intervention period was limited to 14 days. This period was chosen as we wished to examine the effect of the interventions on established disease (i.e. already developed aortic aneurysms) induced over a 28-day AngII infusion period. We believe this is most relevant to the clinical situation. It is plausible that DE may have inhibited aneurysm growth if it was administered over a longer period of time. Finally, conclusions regarding the mechanisms underlying the effect of an intervention using samples from the study end-point have limitations. While we present in *vitro data* in support of *in vivo* findings, analysis of aortic tissue taken from earlier time points may have provided additional mechanistic insight.

In summary, findings of this study suggest an important role for FXa/FIIa in aortic aneurysm growth. Down-regulation of aortic PAR-2, Smad2/3 phosphorylation and MMP-2, and reduced monocyte/macrophage infiltration were demonstrated in a mouse model of aortic aneurysm following administration of the anticoagulant fondaparinux. *In vitro*, FXa stimulated release of PAR-2-mediated MMP-2 from human aortic VSMC. The study identifies a potential for anticoagulants targeting FXa/FIIa in limiting aortic expansion in patients with AAA. Assessment of new generation direct FXa inhibitors that potentially offer more favourable efficacy, safety profile, and more convenient therapy, for AAA growth is warranted. Conclusions regarding the effect of direct FIIa inactivation on aneurysm growth cannot be drawn from the current study and requires further investigation.

## Methods

### FXa and FIIa inhibitors

Fondaparinux sodium was kindly provided by Alchemia Limited, Australia. Chow supplemented with dabigatran etexilate (DE) and matched vehicle was kindly supplied by Boehringer Ingelheim Pharma GmbH & Co KG, Germany. Enoxaparin was purchased from Sanofi-aventis.

### Mouse model

The use of animals for this work conformed to the Guide for the Care and Use of Laboratory Animals (National Institutes of Health, USA). Approval for the mouse studies and experimental work performed was obtained from and in accordance with the James Cook University Animal Ethics Committee (A1480). Male ApoE^−/−^ mice (obtained from Animal Resources Centre, Western Australia) were housed under a 12:12 hour light-dark cycle (relative humidity: 55–60%; temperature: 22 ± 1 °C) and were given standard chow and water *ad libitum*. AAA was induced in 13 week-old male ApoE^−/−^ mice by subcutaneous infusion of AngII (1.0 μg/kg/min) for 28 days, as previously described^44^,^46^. Interventions were initiated 14 days into the AngII infusion period, at which point surviving mice were stratified to vehicle or anticoagulant based upon aortic ultrasound measurement, whereby median and interquartile range SRA diameter were similarly matched between the two groups. Interventions were administered every two days starting at day 15. Five separate studies were performed:

- *Association of aortic FXa with AAA severity and effect of AngII infusion on aortic PAR-2 receptor expression.* In a pilot study, SRA protein was sampled from 8 mice that had received AngII infusion for 28 days. These samples were selected from mice that had a varying response to AngII infusion with SRA diameter ranging from 0.95–2.76 mm. SRA levels of FXa were measured by Western blotting and correlated with SRA diameter. The level of PAR-2 in aortic protein from an additional 12 mice infused with either AngII (n = 6) or saline (n = 6) for 28 days was also determined by Western blotting.
- *Effect of enoxaparin on AAA growth.* Forty mice had AAA induced by AngII infusion and at day 14 surviving mice were randomly allocated to vehicle (n = 16) or enoxaparin (4.5 IU (2 mg/kg); n = 16) via subcutaneous injection every other day for an additional 14 days.
- *Effect of dabigatran on AAA growth.* Thirty mice had AAA induced by AngII infusion and at day 14 surviving animals were randomly allocated to vehicle (n = 11) or intervention chow (7.5 mg DE/gram chow; n = 13) for an additional 14 days. The therapeutic efficacy of the medicated chow has been demonstrated in previous mouse studies^13^,^14^,^15^,^16^,^17^.
- *Effect of fondaparinux on AAA growth.* Thirty mice had AAA induced by AngII infusion and at day 14 surviving animals were randomly allocated to receive vehicle (n = 11) or fondaparinux (300 μg/kg; n = 13) via daily subcutaneous injection for an additional 14 days.

### Assessment of AAA severity

Maximum diameter of the abdominal aorta within the suprarenal region was estimated using ultrasound immediately before commencing and then at days 14 and 28 after starting AngII -infusion. Mice were immobilized by intraperitoneal injection of ketamine (40 mg/kg) and xylazine (4 mg/kg). Ultrasound was performed in B-mode using a MyLa 70 VETXY platform (Esaote, Genoa, Italy) with an LA435 linear transducer (Esaote) at an operating frequency of 12 MHz. Maximum SRA diameter was measured at peak systole using the calliper measurement feature as previously described by our group (1,4). At termination, mice were euthanized by CO_2_ asphyxiation and PBS-perfused dissected aortas (ascending aorta to iliac bifurcation) were placed on a graduated template and digitally photographed (Coolpix 4500, Nikon). The maximum diameter of the aortic arch (aortic valve to the left subclavian artery), thoracic aorta (TA; arch to the diaphragm), SRA, and infrarenal aorta (IRA) were determined from the images using computer-aided analysis (Adobe^®^Photoshop^®^ CS5 Extended version 12, Adobe Systems Incorporated) as previously reported (1). We have previously demonstrated that both ultrasound and morphometry diameters have good inter-observer reproducibility^46^,^47^.

### Atherosclerosis quantification

Sudan IV staining was performed on aortic arch samples to identify intimal atherosclerosis as previously described^46^. In brief, aortic arches were opened longitudinally and fixed in 75% ethanol for 15 minutes. Samples were then stained with Sudan IV solution (0.1% Sudan IV dissolved in equal parts acetone and 70% ethanol) for 10 minutes followed by a 15 minutes 80% ethanol wash. Stained aortic arches were digitally photographed and intimal atherosclerosis areas were measured using computer aided analysis (Adobe Photoshop, USA). We have previously reported good reproducibility of these methods^46^.

### Assessment of aortic proteins

Sample from the SRA were homogenized in the presence of protease inhibitors to obtain protein extracts. Protein concentrations were determined using a Bradford protein assay kit (BioRad, USA) and used for Western blotting or activity assays. For Western blotting samples (25 μg of protein per lane) were separated on a 10% SDS-polyacrylamide electrophoresis gel at 110 volts for 90 minutes then transferred (15 mA, 60 min) to polyvinylidene difluoride membrane (BioRad, USA). Non-reducing conditions were used for FX/FXa blots. The membrane was blocked with 5% non-fat dry milk for 60 min prior to incubation with primary antibodies to FX/FXa (1:1000; Haematologic Technologies Inc., #AMX-9050), PAR-2 (1:1000; Santa Cruz Biotechnology, S-19, #sc-8207), Smad2/3 (1:1000, Santa Cruz Biotechnology, E-20, #sc-6033), or phosphorylated (p)Smad2/3 (1:1000; Santa Cruz Biotechnology, Ser 423/425, #sc-11769-R), overnight at 4 °C. HRP-conjugated IgG (1:1000; DakoCytomation) was used to detect the binding of corresponding primary antibody. Membranes were stripped and re-blotted with anti-GAPDH antibody (1:5000; Cell Signalling, 14C10, #2118) to verify equal loading of protein in each lane. Protein expression was estimated with Western Lightning Chemiluminescence Reagent Plus (PerkinElmer Life Sciences, #NEL103001EA) and quantified by Quantity One software (v 4.6.7; BioRad, USA). Factor X activity (FXa) was determined using the ACTICHROME^®^ Factor X chromogenic activity assay (American Diagnostica Inc., #880) as per manufacturer’s instructions.

### Histology

SRA segments were collected at the end of experiments into optimal cutting compound (ProSciTech), frozen in cold isopentane (Sigma) and stored in −80 °C until required. The renal arteries were used as a landmark to position the tissue and serial sections were taken from the renal arteries to the region of maximal dilatation. Five SRAs were randomly selected from the vehicle, fondaparinux, and dabigatran groups using an online random number generator (https://www.random.org/). Four serial sections of 6 μM thickness were placed on poly-L-lysine coated slides, air dried and subsequently fixed in acetone for 10 min at −20 °C. Adjacent sections were stained with hematoxylin (Polysciences Inc., #24245) and eosin (Polysciences Inc., #09859) (H&E) and mounted in Entellan mounting medium (Electron Microscopy Sciences, #14800) to assess gross morphology. Verhoeff Van Giessen (VVG) staining (Polysciences Inc., #25089) was performed to assess the integrity of SRA elastin. Sections stained with H&E and VVG were photographed using a Nikon Eclipse 50i microscope fitted with a CCD Camera (DSFi1). Digital images were captured to a PC supported with NIS Elements (version F2.30) and analysed as previously described^42^. Histological evaluations were carried out in a blinded fashion in 3–4 sections per sample (mean coefficient of variation (CV) = 2.97%; n = 5).

### Immunohistochemistry

Five SRAs were randomly selected from the vehicle, fondaparinux, and dabigatran groups using an online random number generator (https://www.random.org/) from which serial 6 μM thick cryostat sections were obtained. Briefly, four serial sections were placed on silane-coated slides, were air dried and then fixed in acetone for 10 min at −20 °C. Before processing, the sections were rehydrated with PBS and incubated in 3% H_2_O_2_/0.1% sodium azide/PBS to block endogenous peroxidase. Slides were then incubated in 2% normal goat serum (Vector Laboratories Inc., #S1000) in PBS and endogenous avidin and biotin blocked using a commercial kit (Vector Laboratories Inc., #SP2001), and incubated with anti-monocyte and macrophage antibody (1:200; MOMA-2, abcam, #ab33451) overnight at 4 °C. Sections were rinsed twice in PBS then incubated with biotinylated anti-rat IgG (1:100; Vector Laboratories Inc., #BA-9400) to detect binding of the primary antibody. Sections were washed twice in PBS and incubated in peroxidise-avidin-biotin for 30 min (Vectastain ABC Elite reagent; Vector Laboratories Inc, #PK6100). Slides were incubated in the peroxidase substrate 3,3′-diamminobenzidine (ImmPACT DAB; Vector Laboratories Inc, #SK4105), counterstained in Mayer’s Haematoxylin, dehydrated, cleared in xylene and mounted in Entellan mounting medium (Electron Microscopy Sciences, #14800). Staining of all sections was performed simultaneously using identical reagents and incubation times to avoid batch-to-batch variations. Negative controls were included to identify antibody specificity (omitting primary antibody; see Supplementary Fig. S7). Sections were photographed using a Nikon microscope fitted with a CCD Camera (Diagnostic Instruments, Inc., USA) and Nikon software (BioRad, USA). Identical exposure times and settings were used for all sections. Image analysis was performed on digital tiff images using Adobe Photoshop CS5 Extended software. For each section, the total tissue area and area of staining were measured using the “Selection Tool” and “Record Measurements” functions. Staining was expressed as a percentage of total tissue area (i.e. area macrophage staining/total tissue area × 100). Intra-observer reproducibility was good (CV = 2.68%; n = 5).

### Cell culture

Human aortic VSMCs (Lonza) were maintained in DMEM in T75 flasks at 37 °C in a humidified 5% CO_2_ atmosphere at a density of 2 × 10^5^–1 × 10^6^ cells/ml (passage 5–8). For experiments, VSMCs were seeded at 5 × 10^4^ cells/ml and rendered quiescent for 24 hour by incubation in serum-free medium prior to stimulation. VSMCs were exposed to increasing FXa concentration (0, 5, 15 and 30 nM) over 24 hours. In a follow up experiment, the effect of PAR-1 or PAR-2 antagonist (10 μM) on FXa (30 nM)-mediated Smad2/3 phosphorylation and PAR-2 blockade on MMP-2/9 over 24 hours was examined. Proteins prepared from cell lysates were assayed by Western blotting and zymography for cellular total- and phosphor-smad2/3, and MMP2/9, respectively.

### Zymography

Measurement of MMP2 and MMP9 in conditioned cell culture medium using gelatin zymography was performed as previously described^43^,^44^. Equal-volume samples were run at room temperature on a 10% acrylamide-SDS gel containing 0.5% gelatin. Following several washes in 2.5% (vol/vol) Triton X-100, gels was incubated for 12 hours at 37 °C in 50 m MTris (pH 8) containing 5 mM CaCl2. Bands were visualized in a 10% ethanol/10% acetic acid solution after staining with 0.5% Coomassie blue (R-250), with enzyme activity semi-quantified with densitometric analysis using the ChemiDoc™ imaging system (Bio-Rad Laboratories) and QuantityOne™ 1-D Analysis Software (Bio-Rad Laboratories).

### Real time PCR

QuantiTect^®^ Primer Assays were used to determine gene expression for *Par-1* (QT00119812), *Par-2* (QT02255330), *Mmp2* (QT00116116), and *Mmp9* (QT00108815) in mouse aortic tissue, and, *SMAD2* (QT00004207), *SMAD3* (QT00008729), *MMP2* (QT00088396) and *MMP9* (QT00040040) in human VSMCs using quantitative real time (qPCR) as previously described^48^. The relative expression of these genes in experimental and control samples was calculated by using the concentration-Ct-standard curve method and normalized using the average expression of glyceraldehyde-3-phosphate dehydrogenase (mouse *Gapdh*, QT01658692; human *GAPDH*, QT00079247) for each sample using the Rotor-Gene Q operating software version 2.0.24. The QuantiTect SYBR^®^ Green one-step RT-PCR Kit (Qiagen) was used according to the manufacturer’s instructions with 40ng of total RNA as template. All reactions were independently repeated in duplicate to ensure the reproducibility of the results. For mouse studies, six SRAs were randomly selected from the vehicle, fondaparinux, and DE groups using an online random number generator (https://www.random.org/).

### Statistical Analyses

Data were analysed using GraphPad Prism (version 5) and TIBCO Spotfire S+ (version 8.2). D’Agostino and Pearson test was used to test the normality of the data and parametric or non-parametric tests applied appropriate to data distribution. Comparison of end-point data (Sudan IV staining, WB, IHC) was performed using Mann-Whitney *U* test. The relationship between AAA severity (diameter) and aortic concentration of FX, FXa, FIIa, and fibrin in angII-infused mice was tested using Pearson correlation (r) coefficient. Aortic end-point data for maximum diameter was compared between vehicle and intervention groups by Mann-Whitney *U* test. Mouse data obtained as a function of time (SRA ultrasound) was compared within each group by repeat measures one-way ANOVA, and between vehicle and intervention groups by mixed-effects linear regression. *In vitro* data obtained as a function of concentration or multiple (>2) intervention conditions was compared using Kruskal-Wallis test or ANOVA with Dunn’s or Bonferroni’s multiple comparisons test, respectively, appropriate to data distribution. In all cases P values less than 0.05 were considered significant.

## Additional Information

**How to cite this article**: Moran, C. S. *et al*. Parenteral administration of factor Xa/IIa inhibitors limits experimental aortic aneurysm and atherosclerosis. *Sci. Rep.* **7**, 43079; doi: 10.1038/srep43079 (2017).

**Publisher's note:** Springer Nature remains neutral with regard to jurisdictional claims in published maps and institutional affiliations.

## Supplementary Material

Supplementary Figures

## Figures and Tables

**Table 1:** Effect of fondaparinux and dabigatran administered over 14 days on aortic PAR-1, PAR-2, and MMP2 mRNA within AngII-infused ApoE^−/−^ mice.

Gene	Vehicle	Fondaparinux	DE	P
**n**	6	6	6	
*Par-1*	1.06 (0.82–1.45)	1.16 (0.94–2.04)	1.70 (1.30–1.79)	0.136
*Par-2*	0.86 (0.72–1.09)	0.26 (0.20–0.32)^*^	0.99 (0.87–1.17)	**>0.001**
*Mmp2*	2.19 (1.83–2.59)	0.17 (0.11–0.34)^**†**^	1.39 (0.73–2.82)	**>0.001**
*Mmp9*	1.06 (1.01–1.16)	1.02 (1.00–1.06)	1.04 (1.01–1.09)	0.415

DE, dabigatran etexilate; Data expressed as median (interquartile range) gene expression relative to Gapdh; P, p-value for group comparison by Kruskal-Wallis test;^*^(*P = 0.018) and*^†^(*P = *0.001) compared to vehicle and determined by Dunn’s post-hoc for multiple comparisons.

**Table 2:** Effect of PAR-1 and PAR-2 blockade on FXa-mediated activation of human VSMCs *in vitro* over 24 hours

n	FXa	FXa/PAR1Inh	FXa/PAR2Inh	P
	6	6	6	
**Protein** (*RDU*/*μg protein*)				
Smad2/3 (P/T ratio)	1.64 (1.50–1.88)	1.57 (1.30–1.75)	1.31 (1.22–1.52)^*^	**0.039**
MMP9	0.99 (0.97–1.03)	1.03 (1.01–1.05)	0.96 (0.94–1.01)	0.089
MMP2	1.02 (0.79–1.18)	1.05 (0.97–1.13)	0.36 (0.31–0.44)^**†**^	**0.003**
**Gene Expression** (*relative to GAPDH*)				
*Smad2/3*	0.88 (0.80–1.47)	1.01 (0.93–1.59)	0.61 (0.50–0.74)^**‡**^	**0.002**
*MMP9*	0.64 (0.58–0.76)	0.52 (0.37–0.67)	0.74 (0.55–0.83)	0.110
*MMP2*	0.86 (0.75–1.03)	0.81 (0.69–0.93)	0.57 (0.50–0.64)^**§**^	**0.001**

*Data expressed as median (interquartile range); PAR1Inh, PAR-1 antagonist (SCH79797*)*; PAR2Inh, PAR-2 antagonist (FSLLRY-NH*_*2*_); P/T ratio, ratio of Phosphorylated Smad2/3-to-Total Smad2/3 protein; P, p-value for group comparison by Kruskal-Wallis test;^*^(*P = 0.029*), ^†^(*P = *0.032), ^‡^(P = 0.046), and ^§^(P = 0.005) determined by Dunn’s post-hoc for multiple comparisons.
