# Supplementary material for: Parenteral administration of factor Xa/IIa inhibitors limits experimental aortic aneurysm and atherosclerosis
Source: Sci Rep. 2017 Feb 21;7:43079. doi: 10.1038/srep43079 (PMC5318894; doi:10.1038/srep43079)
Supplement: Supplementary Figures [file srep43079-s1.pdf]

SUPPLEMENTAL MATERIAL

Parenteral administration of factor Xa/IIa inhibitors limits experimental aortic aneurysm and atherosclerosis

Corey S. Moran, Sai-Wang Seto, Smriti Krishna, Surabhi Sharma, Roby J. Jose, Erik Biros, Yutang Wang, Susan K. Morton, Jonathan Golledge.

Supplementary Figures

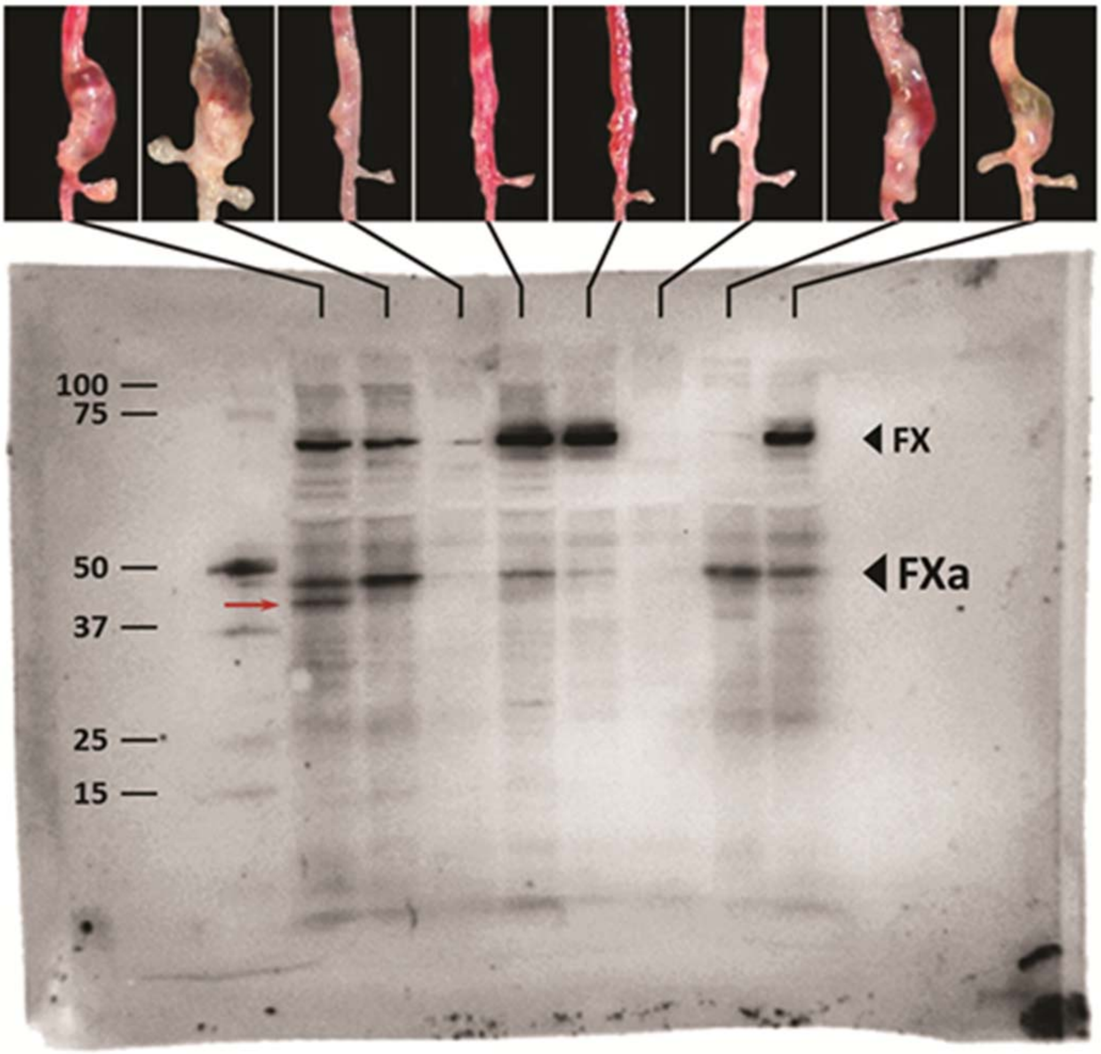

Fig. S1: SRA diameter and FXa in AngII-infused ApoE$^{-/-}$ mice. Western blot showing higher concentration of FXa in SRA with more severe dilatation. Red arrow identifies heavy chain doublet due to conversion of alpha-Xa to beta-Xa by auto-cleavage by alpha-Xa.

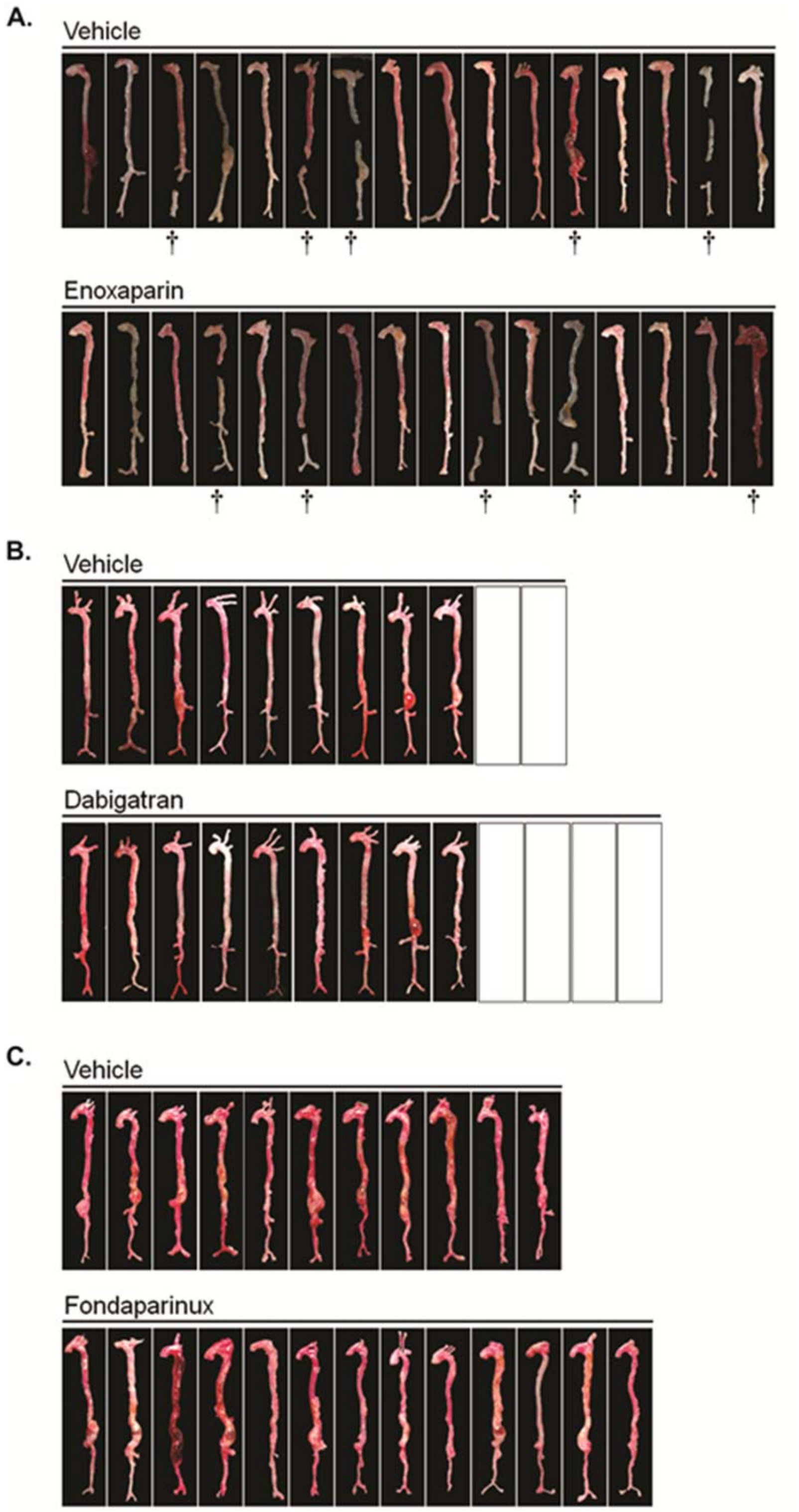

Fig. S2: Effect of FXa and FIIa inhibition on AngII-induced aortic dilatation in ApoE$^{-/-}$ mice. Gross morphology of aortas harvested from mice receiving enoxaparin (**A**; 4.5 IU (2 mg/kg) via subcutaneous injection every other day), dabigatran (**B**; dabigatran etexilate 7.5 mg/gram chow), or fondaparinux (**C**; 300 µg/kg via daily subcutaneous injection) versus vehicle. Administration of anticoagulants commenced 14 days post commencement of AngII infusion (28-day infusion period). † = *fatality due to aortic rupture, aorta successfully retrieved and included in analysis;* □ = *fatality due to aortic rupture, aorta not retrievable and excluded from analysis.*

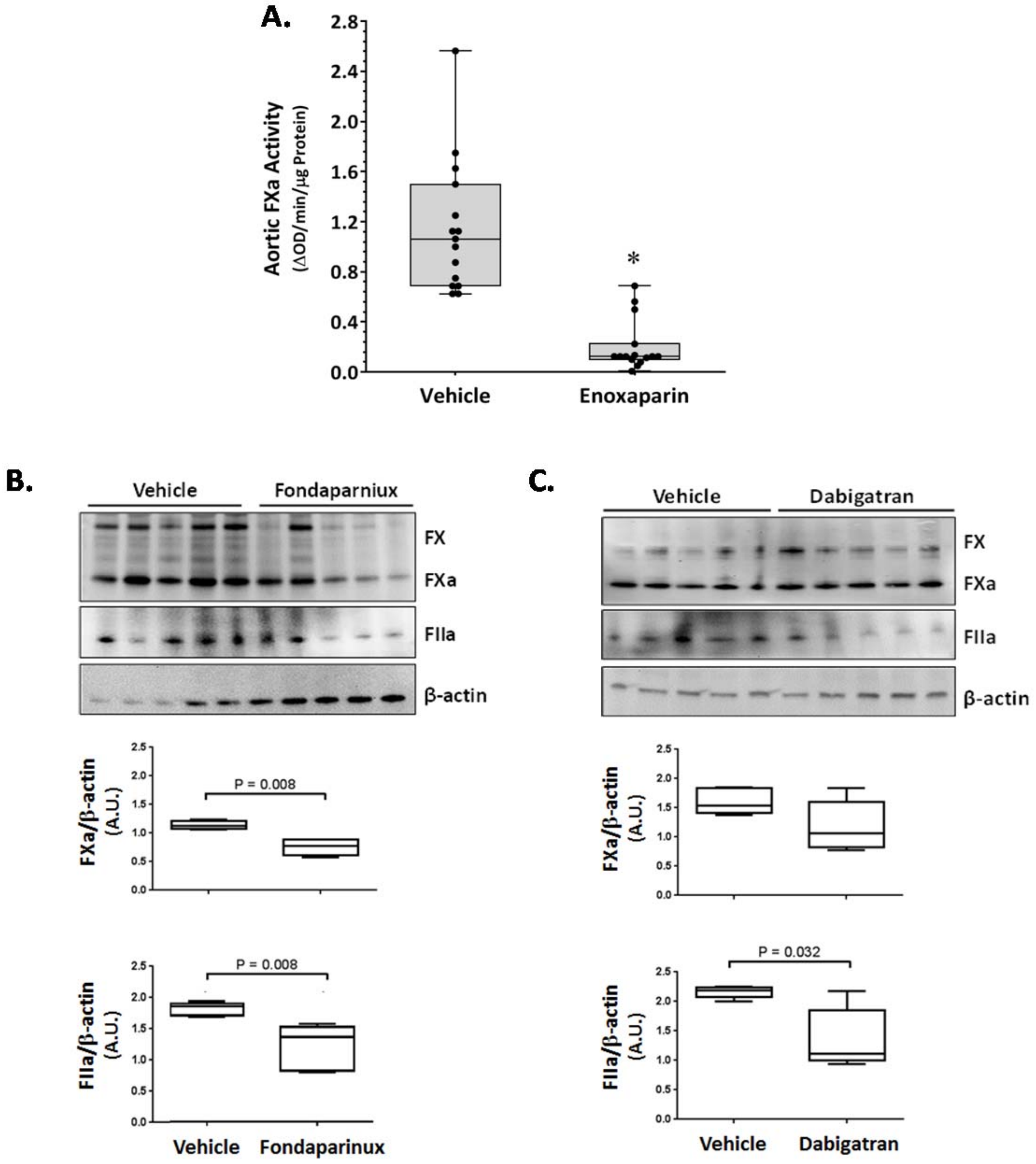

Fig. S3: **A.** FXa activity determined in aortic protein isolate from mice administered enoxaparin compared to vehicle by chromogenic activity assay. Data expressed as median and interquartile range with maximum and minimum data points (whiskers) for change in optical density (OD) per minute per µg protein; *P<0.001 compared by Mann-Whitney U test; n=15. Reduced levels of both FXa and FIIa protein, and FIIa protein alone, in aortic tissue from mice administered fondaparinux (**B**; 300 µg/kg/day) and DE (**C**; 7.5 mg/gram chow), respectively, compared to vehicle as determined by Western blot analysis. Data expressed as median and interquartile range with maximum and minimum data points (whiskers) for FXa/FIIa protein relative to β-actin. P value determined by Mann-Whitney U test. Intervention administered 14 days post commencement of AngII infusion (28-day infusion period). *A.U., arbitrary units/µg protein.*

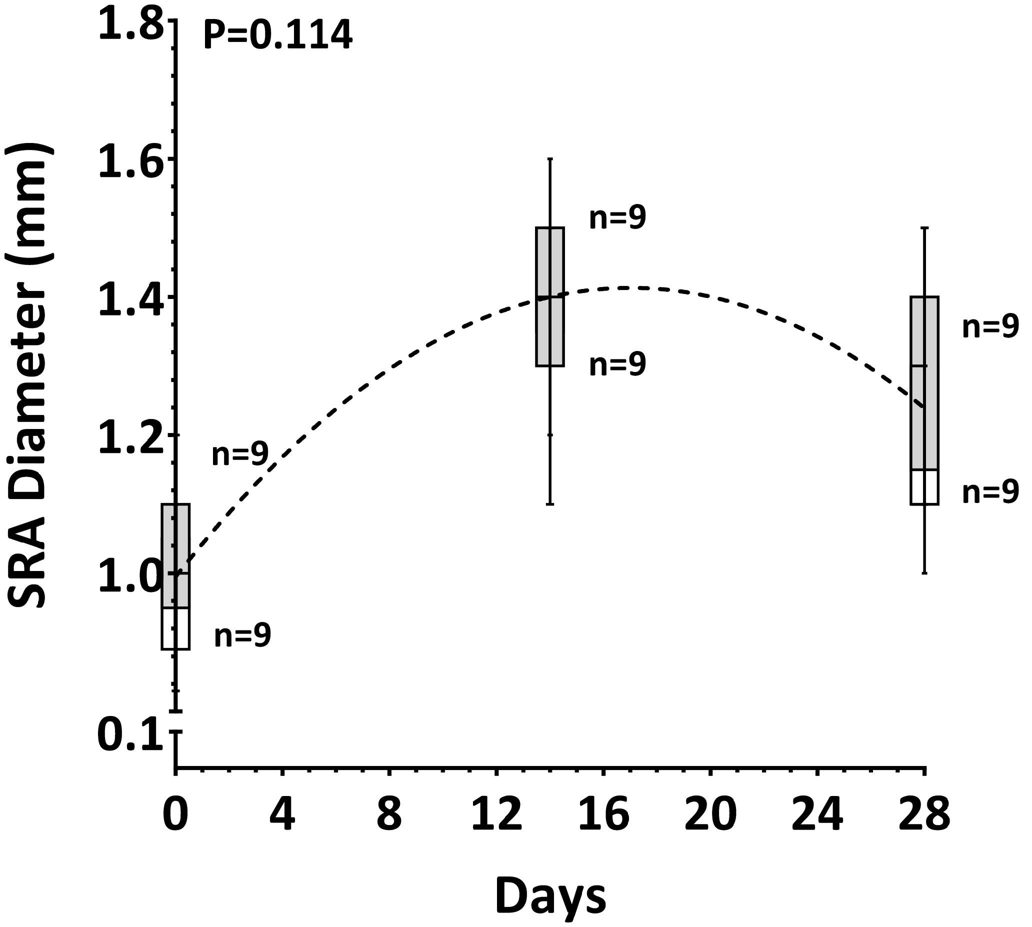

Fig. S4: SRA measured by ultrasound in mice receiving dabigatran (DE)-supplemented chow (7.5 mg/gram chow; white box) or control chow (grey box). Data expressed as median and interquartile range with maximum and minimum data points (whiskers); *P=0.114 for difference between groups by linear mixed-effects; non-linear regression (curve fit) demonstrates one curve for both data sets.

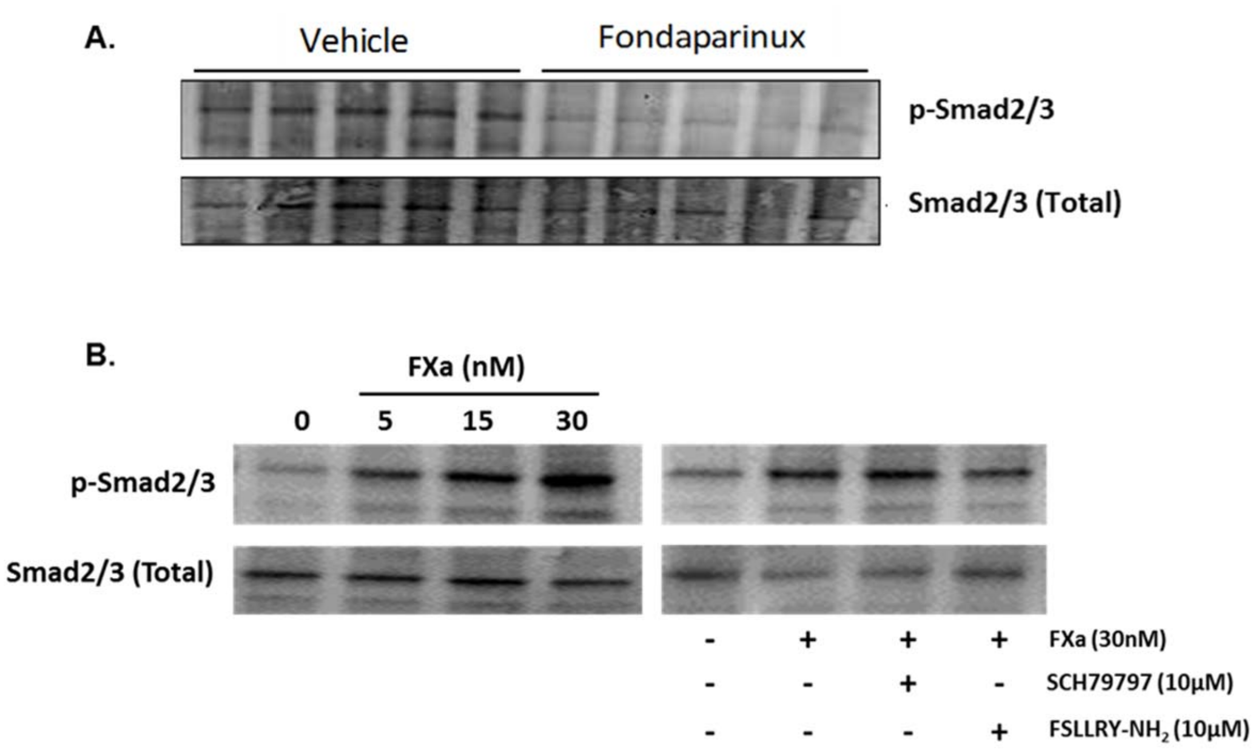

Fig. S5: **A.** Western blot demonstrating similar levels of total Smad2/3 in SRA of ApoE$^{-/-}$ mice administered either vehicle or fondaparinux (300 µg/kg/day) 14 days post commencement of AngII infusion (28-day infusion period), but reduced phosphorylated Smad2/3 (p-Smad2/3) in aortas of mice receiving fondaparinux. **B.** Dose-dependent increase in Smad2/3 phosphorylation in healthy human aortic VSMC exposed to increasing concentration of FXa. Reduced p-Smad2/3 in FXa-stimulated VSMC co-incubated with PAR-2 antagonist (FSLLRY-NH_2) but not with PAR-1 antagonist (SCH79797).

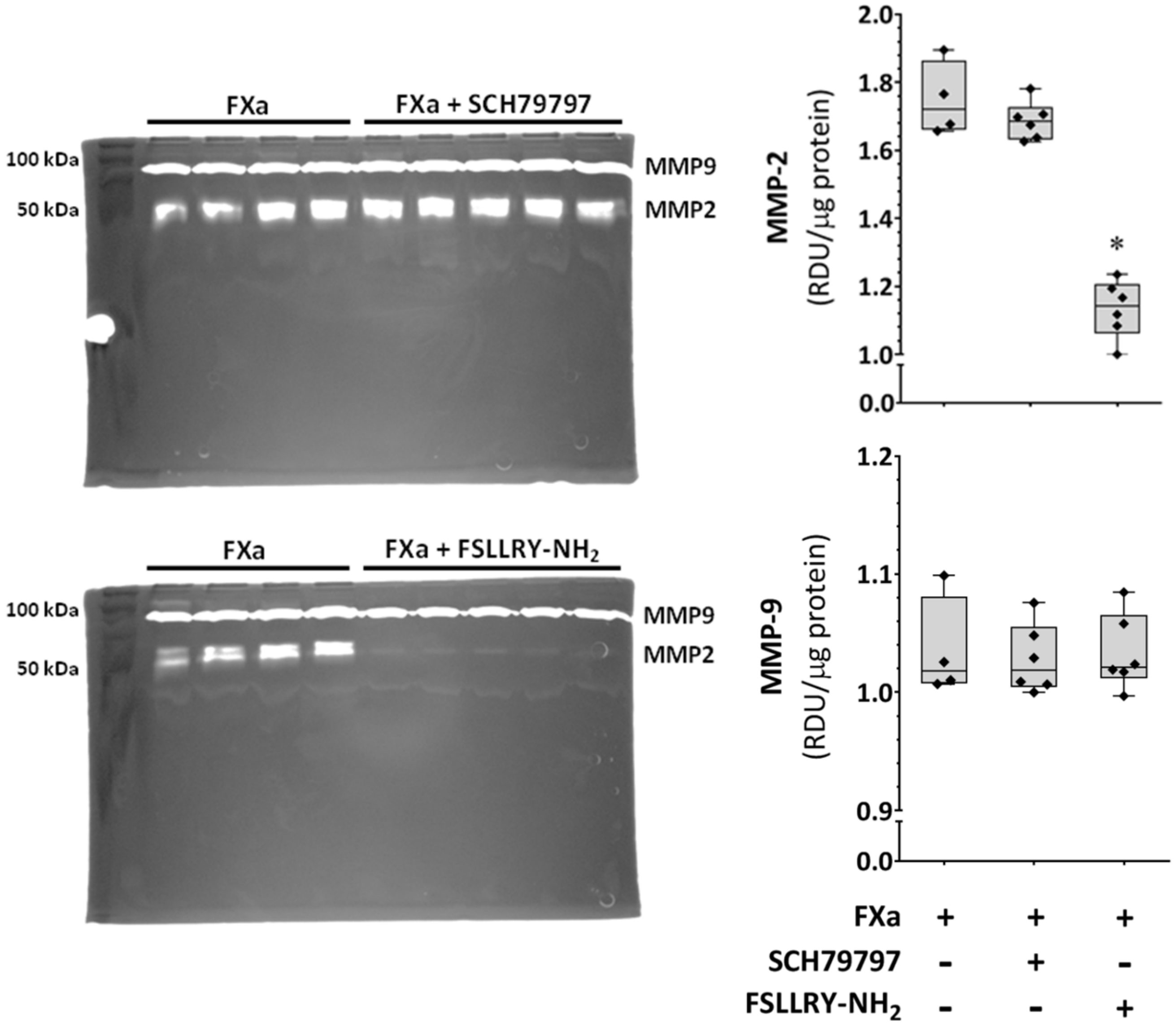

Fig. S6: Zymographic detection of MMP2 and MMP9 in supernatant from healthy human aortic VSMC stimulated with FXa (30 nM, n=4) over 24 hours, demonstrating reduced levels of MMP2 secreted from these cells in the presence a PAR-2 antagonist (FSLLRY-NH2, 10 µM, n=6) compared to a PAR-1 antagonist (SCH79797, 10 µM, n=6). No difference in supernatant level of MMP9 was detected in the presence or absence of either PAR inhibitor. *P=0.002.

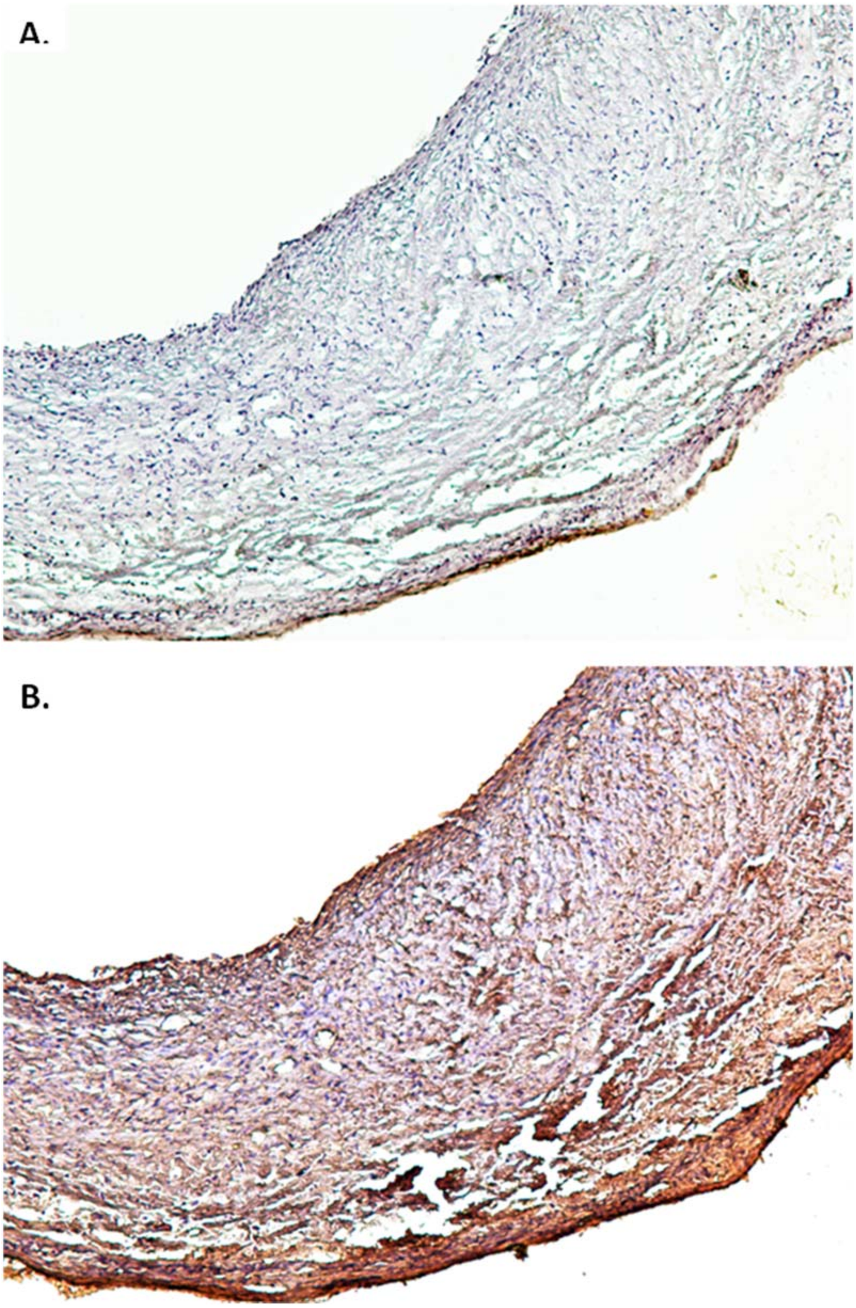

Fig. S7: Representative micrographs demonstrating negative staining (primary antibody omitted; **A**) and positive staining with MOMA-2 (**B**) in 6-μm frozen sections of aortic tissue.
